# Consistency of Eye Coloration Across Different Relationship Partners

**DOI:** 10.1007/s10508-022-02450-0

**Published:** 2022-10-19

**Authors:** Amy V. Newman, Thomas V. Pollet, Kristofor McCarty, Nick Neave, Tamsin K. Saxton

**Affiliations:** Department of Psychology, Northumbria University, Ellison Place, Newcastle-Upon-Tyne, NE1 8ST UK

**Keywords:** Mate choice, Consistency of preferences, Eye color, Evolutionary psychology

## Abstract

Studies have indicated that people are attracted to partners who resemble themselves or their parents, in terms of physical traits including eye color. We might anticipate this inclination to be relatively stable, giving rise to a sequential selection of similar partners who then represent an individual’s “type”. We tested this idea by examining whether people’s sequential partners resembled each other at the level of eye color. We gathered details of the eye colors of the partners of participants (*N* = 579) across their adult romantic history (*N* = 3250 relationships), in three samples, comprising two samples which made use of self-reports from predominantly UK-based participants, and one which made use of publicly available information about celebrity relationship histories. Recorded partner eye colors comprised black (*N* = 39 partners), dark brown (*N* = 884), light brown (*N* = 393), hazel (*N* = 224), blue (*N* = 936), blue green (*N* = 245), grey (*N* = 34), and green (*N* = 229). We calculated the proportion of identical eye colors within each participant’s relationship history, and compared that to 100,000 random permutations of our dataset, using *t*-tests to investigate if the eye color of partners across an individual’s relationship history was biased relative to chance (i.e., if there was greater consistency, represented by higher calculated proportions of identical eye colors, in the original dataset than in the permutations). To account for possible eye color reporting errors and ethnic group matching, we ran the analyses restricted to White participants and to high-confidence eye color data; we then ran the analyses again in relation to the complete dataset. We found some limited evidence for some consistency of eye color across people’s relationship histories in some of the samples only when using the complete dataset. We discuss the issues of small effect sizes, partner-report bias, and ethnic group matching in investigating partner consistency across time.

## Introduction

Much research has sought to illuminate the systematic patterns of variation that underlie individual differences in people’s choice of romantic partner. For example, one such pattern is assortative mating (Štěrbová & Valentova, 2012). Assortative mating, where the partners in a romantic couple demonstrate similarities, has been found in a wide variety of traits, including education levels (Domingue et al., 2014), height (Stulp et al., 2017), facial symmetry (Burriss et al., 2011), attractiveness (Feingold, 1988; Jones et al., 2008; Lee et al., 2008), overall desirability (Conroy-Beam et al., 2019), age and religiosity (Watson et al., 2004), and eye color (Bovet et al., 2012; Laeng et al., 2007; Little et al., 2003; Pennock‐Román, 1984). Another predictor of individual differences in partner choice is family resemblance, particularly in relation to the parent whose gender matches that of the partner, and particularly if the individual has a good relationship with that parent. Thus, research has shown that people’s partners resemble their family members in aspects, such as eye color, hair color, height, body hair, and general facial appearance (Bereczkei et al., 2002, 2004; Bressan, 2020; Bressan & Damien, 2018; DeBruine et al., 2017; Little et al., 2003; Marcinkowska & Rantala, 2012; Saxton, 2016; Saxton et al., 2017; Valentova et al., 2017; Wilson & Barrett, 1987; Wiszewska et al., 2007; see Štěrbová et al. [2017], however, for weaker effects found in same-sex parents and homosexual individuals). That is, to some extent, individual differences in partner choice are systematic and predictable.

Given that people’s own characteristics predict some of the characteristics that they choose in others, we might assume that the qualities that individuals seek in a relationship should demonstrate a degree of stability over time and across different relationship partners: an individual’s selection criteria do not arise arbitrarily, and so neither should they fluctuate arbitrarily. On the other hand, it is conceivable that, following the demise of one relationship, an individual might seek someone dissimilar from their previous partner in the next. Yet so far, there has been little research on within-individual stability in partner choice. This is an oversight, particularly as mate choice can occur several times in an individual’s lifetime. Historically and cross-culturally, sequential and simultaneous mate choice is found regularly within human mating, whether in the form of infidelity, polygyny, polyandry, polyamory, or recoupling after the death of a partner or the dissolution of a relationship (Fisher, 1989, 2016; Fletcher et al., 2015). The question that then arises is the extent to which people choose similar partners across different relationships.

One of the few papers that examined within-individual stability in partner choice (Eastwick et al., 2017) obtained photographs of two or more current/former other-sex partners from 136 university students. Research assistants rated each photograph on a range of physical attributes. The authors found consistency in rated attractiveness, masculinity, and dominance across the photographs of a participant’s current/former partners, irrespective of whether the participant described the relationship as serious or casual. The same researchers also obtained data, collected at three time points across 11 years, in respect of the characteristics of between 2 and 6 partners recorded for 574 focal adolescents / young adults. This dataset provided evidence that participants’ partners were similar to each other on several constructs, most notably IQ, and further that the participants had tended to couple up with people who were similar to them in relation to educational aspirations, religiosity, and IQ. However, the patterns could mostly be explained by social stratification for the characteristics that were assessed (e.g., people with similar IQ are more likely to attend similar schools), or the proximity effect (e.g., Kalmijn, 2006). Finally, the researchers obtained evaluations of the qualities of 145 focal men who had been rated by at least two of their former partners on a popular website. There was no good evidence that the men’s characteristics gave rise to similar evaluations across different relationship partners, in terms of their romantic or sexual qualities, or number of positive and negative qualities. A separate research study (Štěrbová et al., 2018) that also examined the question of similarity across an individual’s partners obtained data from 1048 participants who detailed their previous partners’ hair and eye color. The authors found significant consistency across partners in trait coloration in both long-term and short-term relationships, with small effect sizes. A final study (Štěrbová et al., 2019) found similarities across the relationships of 537 women who reported demographic, physical, and personality characteristics such as weight, facial hair, and eye color, in respect of all of their long-term partners. In that study, although the impact of any single variable tended to be small, the different characteristics summed to a more substantial contribution.

Thus, there is evidence that enduring underlying criteria influence people’s sequential partner selection. However, one cornerstone of psychological research is replication (e.g., Zwaan et al., 2017). In addition, the previous work on physical similarities in sequential partner selection did not directly account for ethnicity, and did not always account for possible mis-remembering in the self-reported data used. It is useful to account for ethnicity in this research area, because apparent consistency in partner characteristics could result from the known tendency for people to select partners of similar ethnic grouping (McClintock, 2010): ethnic groups vary in the frequency of particular physical traits (e.g., the likelihood of having dark hair or eyes), meaning that, for example, an apparent pattern of people consistently selecting dark- or light-eyed partners could arise from a pattern of selecting partners within one’s own ethnic group. Further, it is important to account for possible mis-remembering in self-report data, because mis-remembering may well not be random, and could give rise to apparent consistency in traits across partners. That is, it seems plausible that someone who forgets something of their previous partners’ appearance could be biased to report the same traits for each, erroneously guessing for instance that they all have blue eyes.

Accordingly, in the current study, we set out to examine consistency in partner characteristics across different relationships, while controlling for ethnicity, and including measures to reduce the risk of inaccuracy in the data. As indicated above, the eye color of an individual’s partner is a regular target of study within the context of partner choice. Eye color is relatively easy to measure, salient in interactions, stable across time, and unaffected by variables such as age, health, socioeconomic status, adiposity, stress, or diet, all of which can be apparent in most other physical characters (Bressan & Damian, 2018). Eye color is particularly relevant to relationships, because of the evidence that the diversity of eye color found in European populations has arisen under strong selection pressure (Duffy, 2015), and possibly under sexual selection (Frost, 2006, 2014; Jablonski & Chaplin, 2017). Some research studies have reported evidence for negative frequency dependent selection on eye color: that is, that rarer eye colors are perceived as more attractive (Forti & Young, 2016; Kočnar et al., 2019). In the present study, we chose to focus on eye color as a target of interest in itself, and in addition as a possible proxy for physical appearance more generally. We investigated whether we could replicate findings of consistency in eye color across an individual’s romantic partners, which could not be explained as recollection bias or as a simple outcome of ethnic group matching in partnership formation, in three separate samples.

## Method

### Participants

#### Sample 1

A total of 185 participants (39 males) were recruited through opportunity sampling using social media and on campus at an English university. We did not set out to recruit students explicitly, but it is likely that the majority of the participants were students given how the study was circulated. Our sample size goal was to recruit at least 120 participants within the three-month testing period. Participants had to be aged 18 or over and to have had at least two romantic partners. Participants were aged 18–55 years old (*M* = 23.2 years, *SD* = 7.55 years), and reported between 2 and 21 partners (*M* = 4, *SD* = 3.31), comprising 392 short-term and 352 long-term relationships. The majority of the sample identified as heterosexual (87.6%; bisexual = 8.1%; homosexual = 2.7%; other = 1.6%). While there were no sampling restrictions regarding ethnicity, the sample was mainly White: 91.9%; 2.7% mixed race; 2.7% Asian; 1.6% Black; and < 1% other.

#### Sample 2

A total of 208 participants (75 males) were recruited either through a recruitment website (www.prolific.ac (Palan & Schitter, 2018); n = 150) or via opportunity sampling on social media (n = 58). Although it is not possible to confirm where online participants are sampled from, Prolific recruits participants from within the UK, and the social media sites were targeted to a UK audience. Our sample size goal was to match or exceed the number of participants in Sample 1. Participants had to have had at least two romantic partners and be aged 30–55. Participants from Prolific were reimbursed £1.25 upon completion of the questionnaire. Participants were aged 30–55 years old (*M* = 40.2 years, *SD* = 7.34 years), and reported between 2 and 21 partners (*M* = 6.75, *SD* = 4.27), comprising 801 short-term and 619 long-term relationships. The majority of the sample identified as heterosexual (86.5%; bisexual = 8.7%, homosexual = 4.3%). As in Sample 1, there were no sampling restrictions in terms of ethnicity. The sample was predominantly White (95.6%; < 1% mixed race, < 1% Asian, 1.96% other).

#### Sample 3

Information relating to 185 celebrities (96 males) was obtained from online sources. Some celebrities (actors/musicians) were taken from the IMDB top 100 actors (those with partner information available), and then snowballed to related actors such as famous relatives or cast mates. Our sample size goal was to match or exceed the number of participants in Sample 1. Participants were selected if they were aged over 18 years old, appeared to be of White ethnic origin, and had at least two recorded romantic partners whose identity had been confirmed by the celebrity themselves. Participants were aged 20–65 years old (*M* = 38.0, *SD* = 8.90 years), and had between 2 and 26 (*M* = 5.87, *SD* = 4.12) confirmed partners. Relationships were recorded as long-term (n = 708) if the couple had been together publicly for over six months, or short-term (n = 378) otherwise. The sample was almost entirely heterosexual (97.3%; bisexual = 2.7%). Sexual orientation was coded based on available partner information and/or celebrity interviews. Although the eye color codes used in the analysis were all coded by one researcher, the eye colors were also coded by a second rater for 50 celebrities in order to check reliability, which was moderate to excellent (κ = 0.480, *p* < 0.001 for all eye colors, and κ = 0.847, *p* < 0.001 when split by light and dark eye color).

### Measures and Procedure

The research received ethical approval from the Department of Psychology Ethics Committee at the authors’ institution before data collection commenced. Participants from Samples 1 and 2 were directed to a questionnaire hosted by Qualtrics (www.qualtrics.com), and confirmed that they had had two or more partners (current/previous) in their lifetime. Eligible participants provided their age, gender, ethnicity, and sexual orientation, and were then asked to provide details of all of their sexual/romantic partners in chronological order since the age of 16. For each partner, the participants listed gender, ethnicity, eye color (black, dark brown, light brown, hazel, green, blue green, blue, grey; Little et al., 2003), a rating on a 1–5 scale of the participant’s confidence in their correct recollection of each eye color recorded (where 1 is complete guess, 5 is absolutely certain), and a relationship category (short-term or long-term). Participants were also able to record an eye color of ‘unknown’, or leave the eye color question unanswered; this accounted for 256 data points, and these were excluded from the analysis. Participants were told that short-term relationships were “casual encounters—one night stands, friends with benefits etc.,” while long-term relationships were defined as “committed relationships.” In each sample, participant reports of their confidence in correctly recalling the eye color of each partner ranged from 1–5 (Sample 1: *M* = 4.17, *SD* = 0.68; Sample 2: *M* = 4.15, *SD* = 0.72). To obtain partner eye color information about the celebrities, the lead author consulted well-lit photographs published on fan websites and reports of interviews with celebrities, and recorded the eye color of confirmed romantic partners. Table 1 gives frequency data for the eye color of all of the partners in the study.

**Table 1:** Frequency of partners reported for each eye color, by sample and participant gender, for the complete sample (and in parentheses for those used in the main analysis: i.e. White participants and high-confidence (4 or 5) eye color ratings). Sample 3 participants were all White, and eye color was coded from photographs/interviews

	Dark eyes				Light eyes			
	Black	Dark brown	Light brown	Hazel	Blue	Blue green	Grey	Green
*Sample 1*								
Women	8 (3)	142 (97)	65 (41)	41 (32)	211 (167)	61 (43)	4 (2)	26 (16)
Men	1 (0)	32 (27)	20 (7)	14 (12)	41 (34)	12 (11)	2 (1)	10 (8)
*Sample 2*								
Women	13 (6)	249 (192)	106 (73)	64 (44)	288 (245)	66 (46)	14 (9)	27 (19)
Men	17 (8)	122 (97)	75 (45)	32 (25)	109 (81)	41 (24)	6 (5)	28 (19)
*Sample 3*								
Women	(0)	(159)	(52)	(31)	(134)	(26)	(7)	(64)
Men	(0)	(180)	(75)	(42)	(153)	(39)	(1)	(74)

## Results

All analyses were carried out in R version 4.0.3 (R Development Core Team, 2008). First, we created permutations of the dataset. Each permutation was a version of the dataset where the datapoints, namely the partner eye colors, were randomly transposed. In each permutation, every participant was paired with the same number of partners as in the original dataset (so a participant who listed the eye colors of three partners in the original dataset would still be listed next to the eye colors of three partners in every permuted dataset), and the total quantity of each eye color remained the same (so if there were 200 blue-eyed partners listed in the original dataset, there would also be 200 blue-eyed partners listed in every permuted dataset).

We created 100,000 such permuted datasets. Then, for each participant in every dataset, we calculated the proportion of partners whose eye color made up the largest proportion for that participant. Thus, a participant listed next to four partners (three blue-eyed and one brown-eyed), would have a score of 0.75; a participant who reported four partners (two blue-eyed and two brown-eyed), would have a score of 0.5. Finally, we used a paired-samples *t*-test to compare proportion of matches in the real dataset to each of the 100,000 permuted datasets. As we had a directional hypothesis, we employed one-tailed significance testing. This allowed us to test whether there was consistency in eye color, as exhibited in the array of participants’ real partners, that differed significantly from chance.

The use of simulated datasets meant that we overcame the potential problem that the frequency of colors in the sample might vary (e.g., a majority of brown-eyed people), which could give rise to illusions of systematic partner choices, when in fact consistency of eye color choices across partners could arise simply from a higher prevalence of one color type.

We report the median significance level and effect size using Cohen’s *d* following Westfall (2016). We also analysed long-term and short-term relationships separately, following the same strategy. We ran the analyses both using the original eye color terms, and then separately based upon categorization of the eye colors into light (blue, blue/green, grey, green) or dark (black, dark brown, light brown, hazel) following Little et al. (2003). This reduced the risk that we might conclude that participants consistently select partners with similar eye colors, when in fact participants merely use similar color terms (e.g., one participant describes all of her partners’ eyes as being ‘light brown’, while another describes all of his partners’ eyes as being ‘hazel’). The code and data are available on the OSF, along with additional figures and analyses not reported here (e.g., permutation based significance tests rather than *t*-tests).

For our main analysis, detailed in Table 2, we restricted the dataset to participants of White ethnicity, and in relation only to partners whose eye color was recalled with a confidence level of “4” (“I’m pretty sure”) or “5” (“absolutely certain”). Following Stulp et al., (2013a, b)), we present the median *p* value (one-tailed), median effect size, and the percentage of permutations where the original dataset had a significantly higher proportion of matches. As shown in Table 2, there was no evidence for consistency in eye color across different partners in any of these samples, irrespective of whether the eye colors were categorized as the original color terms, or expressed in terms of dark vs light eye colors.

**Table 2:** Summary of *t*-tests to determine whether eye color was significantly more consistent across partners in the real than control (permuted) datasets, for participants of White ethnicity, in respect of partners for whom we had high confidence in the eye color report

Sample	Eye color categorization	Relationship type (total number of partners)	Median one-tailed *p-*value [95% CIs]	Median *d* [95% CIs]	% significant (*p* < .05, one-tailed)
Sample 1	All colors	All relationships (N = 501)	*p* = .22 [.02, .68]	*d* = 0.06 [< 0.01, 0.16]	8%
		Short-term (N = 206)	*p* = .46 [.07, .88]	*d* = 0.03 [< 0.01, 0.11]	1%
		Long-term (N = 293)	*p* = .10 [< .01, .47]	*d* = 0.10 [0.01, 0.19]	28%
	Dark/Light	All relationships (N = 501)	*p* = 0.34 [.04, 0.83]	*d* = 0.05 [< 0.01, 0.17]	4%
		Short-term (N = 206)	*p* = .81 [.29, .99]	*d* = 0.09 [< 0.01, 0.24]	< 1%
		Long-term (N = 293)	*p* = .11 [< .01, .24]	*d* = 0.12 [< 0.01, 0.24]	26%
Sample 2	All colors	All relationships (*N* = 938)	*p* = .27 [.04, .69]	*d* = 0.05 [< 0.01, 0.14]	< 1%
		Short-term (*N* = 429)	*p* = .09 [< .01, .42]	*d* = 0.10 [0.02, 0.18]	32%
		Long-term (*N* = 509)	*p* = .08 [< .01, .41]	*d* = 0.11 [0.02, 0.19]	33%
	Dark/Light	All relationships	*p* = .39 [.05, .86]	*d* = 0.04 [< 0.01, 0.14]	< 1%
		Short-term	*p* = .21 [.02, 0.71]	*d* = 0.07 [< 0.01, 0.19]	10%
		Long-term	*p* = .25 [.02, .76]	*d* = 0.06 [< 0.01, 0.18]	9%
Sample 3	All colors	All relationships (*N* = 1,033)	*p* = .28 [< .01, .68]	*d* = 0.06 [< 0.01, 0.16]	2%
		Short-term (*N* = 359)	*p* = .08 [< .01, .44]	*d* = 0.10 [0.02, 0.18]	34%
		Long-term (*N* = 674)	*p* = .37 [.06, .82]	*d* = 0.04 [< 0.01, 0.13]	2%
	Dark/Light	All relationships	*p* = .27 [.03, .75]	*d* = 0.07 [< 0.01, 0.19]	6%
		Short-term	*p* = .32 [.03, .83]	*d* = 0.05 [< 0.01, 0.16]	5%
		Long-term	*p* = .39 [.05, .86]	*d* = 0.05 [< 0.01, 0.16]	3%

**Fig. 1:**
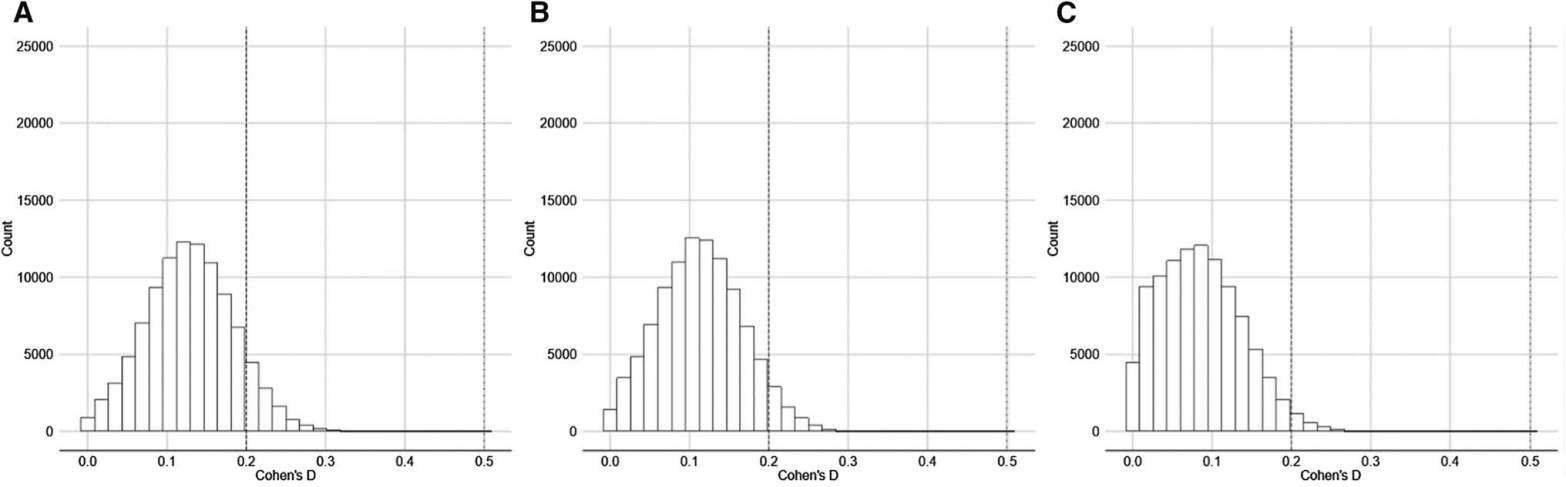
Cohen’s d distributions for analyses including all eye colors (**A** Sample 1, **B** Sample 2, **C** Sample 3). 0.2 Reference line for small effect

**Fig. 2:**
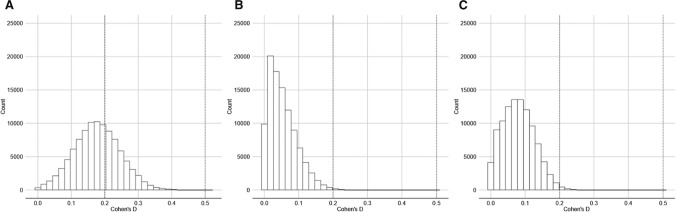
Cohen’s *d* distributions for analyses categorising eye color into dark and light (**A** Sample 1, **B** Sample 2, **C** Sample 3) 0.2 Reference line for small effect

We also ran the analyses in relation to the complete dataset: that is, by including all participants irrespective of ethnicity, and irrespective of the confidence that participants had in their recollection of partner eye color. The results of those analyses are given in Table 3. In this analysis, some evidence for consistent eye color choices across romantic relationships came from Sample 1, when eye colors were categorized into dark or light. However, when short-term and long-term relationships were considered separately, it became clear that this effect was driven by the long-term relationship data. Other evidence for consistent eye color choices across sequential romantic relationships came from Sample 2, when all eye colors were considered (instead of being categorized into dark vs light), in relation to long-term relationships only. However, when we excluded all participants who did not categorize themselves as White (analysis on OSF), only the effects from Sample 2 were statistically significant. Figures 1 and 2 illustrate the distributions of effect size.

**Table 3:** Summary of *t*-tests to determine whether eye color was significantly more consistent across partners in the real than control (simulated) datasets, for all participants, in respect of all reported eye colors

Sample	Eye color categorization	Relationship type (total number of partners)	Median one-tailed *p-*value [95% CIs]	Median *d* [95% CIs]	% significant (*p* < .05, one-tailed)
Sample 1	All colors	All relationships (690)	*p* = 0.08, [0.01, 0.42]	*d* = 0.13, [0.02, 0.24]	36%
		Short-term (347)	*p* = 0.18, [0.01, 0.64]	*d* = 0.06, [0.01, 0.14]	13%
		Long-term (343)	*p* = 0.06, [0.01, 0.37]	*d* = 0.11, [0.03, 0.20]	44%
	Dark/Light	All relationships	*p* = 0.03, [0.01, 0.33]*	*d* = 0.17, [0.04, 0.31]	60%
		Short-term	*p* = 0.45, [0.06, 0.90]	*d* = 0.06, [0.01, 0.14]	2%
		Long-term	*p* = 0.04, [0.01, 0.32]*	*d* = 0.17, [0.04, 0.29]	59%
Sample 2	All colors	All relationships (1257)	*p* = 0.05, [0.01, 0.31]	*d* = 0.14, [0.04, 0.23]	47%
		Short-term (657)	*p* = 0.11, [0.01, 0.49]	*d* = 0.08, [0.01, 0.16]	24%
		Long-term (600)	*p* = 0.01, [0.01, 0.14]*	*d* = 0.18, [0.09, 0.27]	85%
	Dark/Light	All relationships	*p* = 0.20, [0.02, 0.68]	*d* = 0.08, [0.01, 0.20]	11%
		Short-term	*p* = 0.18, [0.01, 0.65]	*d* = 0.08, [0.01, 0.19]	14%
		Long-term	*p* = 0.09, [0.01, 0.52]	*d* = 0.12, [0.01, 0.24]	32%

*p < .05

## Discussion

Popular culture leads us to believe that individuals have a “type,” a predilection for certain physical characteristics that is apparent across sequential partner choice. Indeed, as set out in the Introduction, there is a good theoretical justification for anticipating some degree of consistency in eye coloration (and also in general physical appearance) across partners, given that people prefer or select partners who resemble themselves and their parents in relation to physical characteristics such as eye color (Bressan, 2020; Bressan & Damien, 2018; Little et al., 2003; Saxton, 2016; Štěrbová et al., 2018, 2019; Wilson & Barrett, 1987). Accordingly, we set out to investigate whether consistency in choice of physical characteristics (specifically, eye color) was evident across an individual’s relationship history. We analyzed the data using the original eye color terms, and then again when eye color was categorized into “light” (blue, blue/green, grey, green) and “dark” (black, dark brown, light brown, hazel) to reduce the risk that apparent eye color consistency across relationships could be due merely to idiosyncrasies in color term usage, following previous work (Little et al., 2003).

In our main analysis, we restricted participants to those of White ethnicity so as to control for ethnic group matching, and also only made use of eye colors where we had a good degree of confidence in their accuracy. We did not find any substantial evidence that people’s partners exhibited similar eye colors. However, in our secondary analysis, where we included all participants irrespective of ethnicity and confidence in eye color recollection, we found some evidence for consistent eye color choices across relationship partners in some samples. Specifically, some evidence arose from Sample 1 when their partners’ eye colors were categorized as dark or light. There, we found consistency of eye color across long-term relationship partners: Individuals were more likely to select either more dark-eyed or more light-eyed long-term relationship partners than would be expected by chance. Other evidence for eye color consistency across long-term relationship partners arose from Sample 2, when the complete range of eye colors was considered (instead of being categorized as dark or light). This suggestion that eye color consistency may be more apparent across long-term than short-term relationships is consistent with evidence that individuals are more likely to lower their requirements in short-term relationship contexts (Kenrick et al., 1990, 1993; Li & Kenrick, 2006; Stewart et al., 2000), although the diminished likelihood of accurate recall of correct eye color for short-term compared to long-term relationships could also be a factor.

Given our mixed pattern of results, coupled with findings of a small degree of consistency in eye coloration across partners revealed in previous research (Štěrbová et al., 2018, 2019), we would suggest that any consistency in eye coloration across partners would be of small effect size. Small effects are typical of findings within social psychology (Richard et al., 2003). We were interested in eye color both in its own right, and also as a possible proxy of general physical appearance, and it is possible that measuring additional physical appearance variables could make manifest greater consistency across a set of partners (see Štěrbová et al., 2019). On the other hand, it is also possible that the estimated effect sizes have been inflated by biased misremembering and ethnic group matching, both of which could drive up apparent consistency across relationship partners.

Future work should seek to further disentangle these potential sources of error. Researchers have sought to overcome inaccurate recollection of eye color by asking participants to omit answers unless they were confident in their answers (Štěrbová et al., 2018), or to rate their confidence in their recollection on a 1–5 scale (our study). In our study, on average, people believed their recollection to be good, and indeed, previous research that asked students to state their parents’ hair and eye color, and then subsequently to contact their parents to ask for the parents’ own description of their hair and eye color, found a high degree of consistency between the two sets of reports (Saxton, 2016).

Our study benefits from sampling within and outside the standard undergraduate cohort. Sample 1 recruited around student groups, and consisted of predominantly younger people (only 25 participants were over 30 years old), and so an older group was selected for Sample 2 (aged 30–55), allowing us to check how partner appearance consistency might change with age, and also to interrogate a sample with a longer adult history and hence a greater likelihood of furnishing more data points (prior partners). In addition, our sample was UK based, which benefits from high eye color diversity (Walsh et al., 2012). Finally, Sample 3 consisted of celebrities. On the one hand, celebrities are typically considered very desirable partners, with access to many dating pools, and so they are people who might be freer to realize their physical preferences than other groups. Indeed, a recent 45-country study of over 14,000 participants found that people of higher mate value are better able to realize their preferences in a partner (Conroy-Beam et al., 2019), while another large sample found that, while men and women preferred partners with the eye color of their other-sex parent, only attractive women converted their preferences into actual relationship choices (Bressan, 2020; Bressan & Damian, 2018). On the other hand, celebrity dating may also be hampered by particular constraints, such as media scrutiny, lack of privacy, and the pressures of being a “brand.” In any case, our celebrity sample did not provide any evidence for consistency in eye color across relationships.

Eye color is unlikely to be a priority in partner choice (Štěrbová et al., 2018), and yet there is good evidence for strong selection pressure on the evolution of eye color variety (Duffy, 2015), with some researchers suggesting that the diversity of eye color seen particularly in European populations has been sexually selected, that is, has arisen under the selection pressure of human mate choice (Frost, 2006, 2014). Individuals’ idealized preferences for eye color might be less apparent from a dataset of actual relationships, in which people may not be able to realize all their partner preferences (Baldauf et al., 2009; Bressan, 2020). Time constraints may mean that people settle for less than perfect partner attributes (Cotton et al., 2006). The existence of a partner who matches your preferences, and is available in the face of potential competition from other individuals, means that very few people will be able to obtain a partner who fulfils all of their preferences (Conroy-Beam & Buss, 2016). Alternatively, we might see individuals looking for a new partner with similar characteristics to their prior if the relationship was good, but looking for a change if the relationship was less healthy, in order to diversify. Future research should investigate the quality of past relationships in regards to similarity among partners.

Our data present an apparent paradox. Previous research has indicated that people’s parents and partners have similar eye color, which would indicate that people should be more likely to couple up with partners who have similar eye color across multiple relationships, but our data do not point to this as a sizable effect. To resolve this paradox, we might suggest that individuals’ preferences for eye color are most apparent in the relationships most likely to have been captured in previous studies, which are those ones that last the longest amount of time, and are thus more likely to be picked up in cross-sectional sampling. There is some limited support for this point in that our results were clearest in relation to the long-term rather than short-term relationships.

Various explanations have been given for the tendency of people to partner with those who resemble them. At the proximate level, people are more likely to encounter others who are similar, due to social, cultural, and ethnic geographic stratification, together with the influence of local environments on physical traits. Further, physical similarity may engender feelings of trust (DeBruine, 2005). Heritability of partner choice could also be a factor, with some evidence suggesting an “imprinting-like” effect can be seen in humans (e.g., Bereczkei et al., 2002, 2004; Zietch et al., 2011; however cf. Marcinkowska & Rantala, 2012). At the ultimate level, the suggestion has been put forward that coupling with someone who is somewhat physically similar could support reproduction. Reproducing with someone who is genetically similar to you (i.e., closely related) can reduce the health of offspring due to recessive genetic disorders, but equally, reproducing with someone who is genetically very different from you could disrupt useful locally adapted genetic complexes and useful genetic heterozygosity, and so ‘optimal outbreeding’ could give rise to the highest number of offspring (see e.g., Bateson, 1983; Edmands, 2007). Indeed, a study of all couples in Iceland found that the highest number of grandchildren were born to those who were related at about the level of third or fourth cousin (Helgason et al., 2008). However, while it is easy to create evolutionary explanations of patterns—evolutionary theorising is powerfully generative, after all—determining whether they constituted a realistic selection pressure for a behavior to evolve is a different matter.

In conclusion, we found some limited evidence for consistency in eye coloration across romantic relationships (perhaps long-term more than short-term relationships), of small effect size. Future work might seek to further investigate consistency in appearance across partners, expanding this work to consider variables beyond eye color, while continuing to account for ethnic group matching and the accuracy of the reported variables, and to investigate the possible influence of one’s sentiment towards one’s former partner in selecting (or not) a similar partner in future.

## Data Availability

https://osf.io/fstv9/?view_only=3e49818da3f943a3ad1cf355e4d7b3e1

## Code Availability

https://osf.io/fstv9/?view_only=3e49818da3f943a3ad1cf355e4d7b3e1
